# Expression of Semaphorin 3F and Its Receptors in Epithelial Ovarian Cancer, Fallopian Tubes, and Secondary Müllerian Tissues

**DOI:** 10.1155/2009/730739

**Published:** 2009-11-01

**Authors:** Christina D. Drenberg, Sandra Livingston, Ren Chen, Patricia A. Kruk, Santo V. Nicosia

**Affiliations:** ^1^Department of Pathology and Cell Biology, University of South Florida, Tampa, FL 33612, USA; ^2^Office of Clinical Research, University of South Florida, Tampa, FL 33612, USA; ^3^H. Lee Moffitt Cancer Center and Research Institute, Tampa, FL 33612, USA; ^4^Department of Oncological Sciences, University of South Florida, Tampa, FL 33612, USA

## Abstract

While semaphorins and their receptors appear to play a role in tumor carcinogenesis, little is known about the role of semaphorin 3F (S3F) in epithelial ovarian cancer (EOC) development. Therefore, we sought to determine the clinical relationship between S3F and its receptors, neuropilin-2 (NP-2) and neuropilin-1 (NP-1) with EOC progression. We analyzed the immunohistological expression of S3F, NP-2, and NP-1 in clinical specimens of normal ovaries (N), benign cystadenomas (Cy), well-differentiated adenocarcinomas (WD), poorly-differentiated adenocarcinomas (PD), inclusion cysts (IC), paraovarian cysts (PC), and fallopian tubes (FT). Tissue sections were evaluated for staining intensity and percentage of immunoreactive epithelia. We found that expression of S3F and NP-2 decreased while NP-1 expression increased with EOC progression. Interestingly, we also found elevated expression of S3F, NP-2, and NP-1 in epithelia of ICs, PCs, and FT. Our findings indicate that loss or deregulation of semaphorin signaling may play an important role in EOC development.

## 1. Introduction

Epithelial ovarian cancer (EOC) is the most lethal and the second most commonly diagnosed gynecological malignancy. It is estimated that in 2009, it will strike over 21000 women seventy percent of whom will be first diagnosed at advanced stages and will die within five years [1]. In general, EOC is characterized by poor prognosis due to lack of early symptoms, which contributes to advanced stage of disease at presentation, and by the absence of accurate screening methods to detect early stages of the disease. The origin of this malignancy has been traditionally attributed to the ovarian surface epithelium (OSE). However, alternative theories to a coelomic origin attribute the source of EOC to primary or secondary Müllerian system derivatives such as the fimbriated end of the fallopian tube and paraovarian vestigial structures, respectively [2–4]. The Müllerian system theory would explain why epithelial ovarian neoplasms present as morphological variants of fallopian tube (serous adenocarcinoma), uterus (endometrioid), or endocervix (mucinous adenocarcinoma) epithelia without requiring an intermediate metaplastic step [4]. Independently of its cell of origin, the pathogenesis of this most lethal gynecologic malignancy is, however, not well understood. 

 Semaphorins are a large family of transmembrane, secreted, or glycosylphosphatidylinositol (GPI) anchored, proteins involved in axon guidance and growth cone collapse through interaction with their receptors, the neuropilins and plexins [5]. There are eight classes of semaphorin genes all of which are characterized by a conserved 500 amino acid, cystine-rich Sema domain, which mediates binding specificity and is necessary for signaling [5]. Plexins are transmembrane receptors that form complexes with neuropilin transmembrane receptors and mediate signal transduction following binding to a semaphorin [6]. Additional biological functions for semaphorins and their receptors include regulation of angiogenesis as well as tumor progression and metastasis [5, 7]. 

 With regard to angiogenesis, the class 3 semaphorins are of interest since members of this class have demonstrated either pro- or antitumorigenic functions. Class-3 semaphorins are unique in that they directly bind neuropilin homo- or heterodimeric receptors and are unable to bind directly to plexins [8–11]. However, signaling is regulated through an oligomeric complex involving a neuropilin dimer and one of four type-A plexins [6, 12–14]. Interestingly, neuropilins also function as coheteroreceptors with vascular endothelial growth factor receptors for vascular endothelial growth factor (VEGF), whose over expression contributes to tumor growth and metastasis [15]. Of interest is semaphorin 3F (S3F), a class 3 secreted protein which plays a critical role during neural development in both the peripheral and central nervous systems through interaction with its high affinity receptor NP-2 and low-affinity receptor NP-1 [13]. S3F has also been shown to inhibit angiogenesis by decreasing blood vessel density and through competition with VEGF for a shared receptor complex [16, 17]. Specifically, S3F induces a poorly vascularized, encapsulated, non-metastatic phenotype through chemorepulsion of endothelial cells in melanoma [18]. In breast cancer, S3F disrupts intercellular contacts of MCF7 breast cancer cells through delocalization of E-cadherin and *β*-catenin [7]. Further, S3F and VEGF demonstrate opposing effects for cell attachment and spreading [19], as well as migration [20]. 

S3F loss or delocalization has been shown to correlate with advanced tumor stage in a number of cancers including lung and breast [21]; however, a correlation between S3F and tumor stage, grade, and histological subtype remains to be demonstrated in ovarian cancer. In order to begin to better understand epithelial ovarian carcinogenesis, we sought to determine the clinical relationship between S3F and EOC progression. Therefore, we analyzed the immunohistochemical expression of S3F and its two receptors NP-1 and NP-2 in clinical specimens. 

## 2. Materials and Methods

### 2.1. Tissue Specimens

With institutional approval, 44 specimens were retrieved from the tissue bank at H. Lee Moffitt Cancer Center and Research Institute. Serial 4-5 *μ*m sections were hematoxylin and eosin stained and classified according to FIGO criteria (International Federation of Gynecology and Obstetrics) as normal ovaries (N, *n* = 12), benign serous cystadenomas (Cy, *n* = 10), well-differentiated serous cystadenocarcinomas (WD, *n* = 4), poorly differentiated serous cystadenocarcinomas (PD, *n* = 6) and fallopian tubes (FT, *n* = 4). Three of 4 WD carcinomas were late stage (III-IV) whereas all PD specimens were of late stage. We also evaluated epithelia of inclusion cysts (IC, *n* = 6) and paraovarian cysts (PC, *n* = 2) from patients with otherwise normal ovaries and fallopian tubes.

### 2.2. Immunohistochemistry

Immunohistochemical staining was performed on serial paraffin-embedded sections by the horseradish peroxidase (HRP) conjugated system using a Dako Autostainer Plus (Dako NorthAmerica, Inc., Carpinteria, CA). Microwave antigen retrieval was achieved using 10x Antigen Retrieval AR-10 (Tris) (BioGenex, San Ramon, CA) or 10mM citrate buffer for 17 minutes. Sections were rinsed twice with deionized water, washed in Tris buffered saline (TBS)/Tween for 5 minutes and immunostained on the Dako Autostainer with the following: rabbit antiS3F polyclonal antibody (Chemicon, Billerica, MA) at 1 : 50 for 1 hour at room temperature, rabbit antineuropilin-1 polyclonal antibody (ECM Biosciences, Versailles, KY) at 1 : 200 overnight at 4°C, and the mouse antineuropilin-2 monoclonal antibody (Santa Cruz Biotechnology, Inc., Santa Cruz, CA) at 1 : 75 for 1 hour at room temperature. Secondary antibodies for S3F and NP-2 were Vector Elite ABC Peroxidase, using rabbit IgG and Goat IgG, respectively; DAB was the chromogen. The secondary antibody for NP-1 was EnVision + Peroxidase polymer. Endogenous peroxidase was blocked with 3% aqueous hydrogen peroxide. Sections were counterstained with modified Mayer's hematoxylin. 

Immunostaining of S3F, NP-1, and NP-2 was evaluated by two independent observers (SVN and CD) and scored based on staining intensity from 1 to 3 (0; negative; 1; weak; 2; moderate; and 3; strong) and percent of positive epithelial cells (1; 1–10%; 2; 10–50%; and 3; >50%). Cellular localization of S3F, NP-1, and NP-2 was also assessed. To confirm the specificity of the antibodies, non-immune rabbit IgG and goat IgG were used as negative controls in place of primary antibodies for tissue specimens. Specificity was further confirmed by Western blot analyses of cell lysates and visualization of the corresponding protein bands at the appropriate molecular weights for the respective antibodies (data not shown).

### 2.3. Statistical Analyses

Statistical analysis of staining for S3F, NP-1, and NP-2 among clinical samples was analyzed by Spearman rank correlation and Fisher exact test for differences in staining intensity and histological type. ANOVA analyses were performed to determine significant differences in percentage of positively stained epithelia between N, N combined with FT, Cy, and cancer (WD combined with PD) groups. Spearman and Fisher exact tests were performed with SAS version 9.2 (SAS Institute, Cary, NC) and ANOVA tests were performed with Microsoft Excel (Microsoft, Redmond, WA). *P*-values < 0.05 were considered statistically significant.

## 3. Results

### 3.1. S3F Expression Decreases with Epithelial Ovarian Cancer Progression

**Figure 1:**
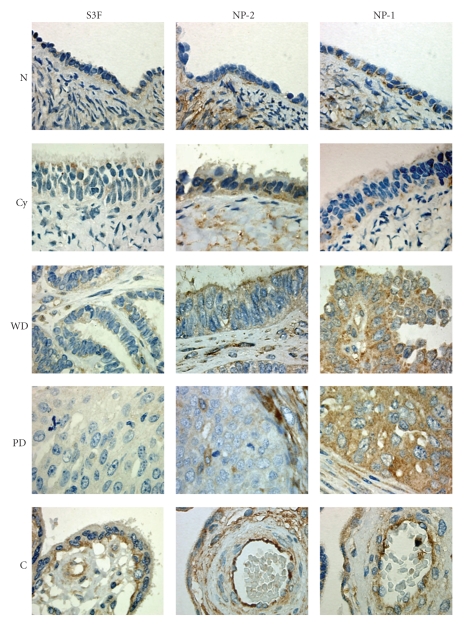
S3F expression decreases while NP-1 increases with epithelial ovarian cancer progression. Representative illustrations of immunohistochemical staining of normal (N), serous cystadenoma (Cy), well-differentiated (WD) and poorly differentiated (PD) serous adenocarcinomas) for S3F, NP-2, and NP-1. Placental tissue was used for positive control (C) and arrow indicates expression of S3F by endothelial cells. Primary antibodies were replaced with non-immune serum in negative control sections (inset). Original magnification: 400×.

**Figure 2:**
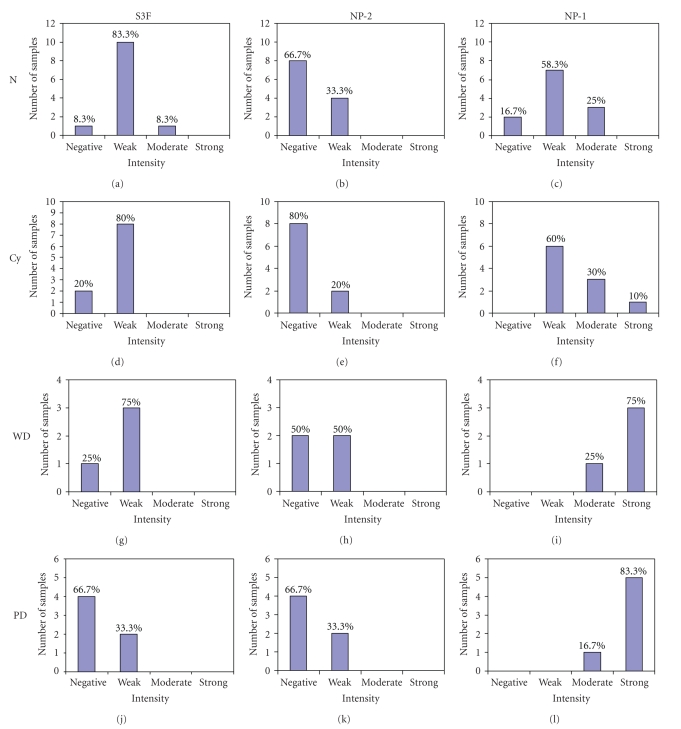
Graphical depiction of S3F, NP-2, and NP-1 expression with epithelial ovarian cancer progression. Immunohistochemically stained sections of normal (N), serous cystadenomas (Cy), well-differentiated (WD) and poorly differentiated (PD) serous adenocarcinomas were evaluated for expression of S3f, NP-2 and NP-1 and scored as negative, weak, moderate, or strong as described in Materials and Methods.

When all histological subtypes were considered, the expression level of S3F in epithelial cells was relatively weak and decreased with tumor progression. We found a significant inverse correlation between S3F staining intensity and histology where 83.3% (10/12) of N, 80% (8/10) of Cy, and 75% (3/4) of WD specimens expressed weak S3F staining, whereas the majority of PD specimens, 67% (4/6), completely lacked S3F expression (Figures 1 and 2) (*P* < .0001). No differences were observed as function of stage. Interestingly, when we evaluated the percentage of positive epithelia in the sections expressing S3F a significantly higher percentage of normal OSE was immunoreactive compared to Cy (*P* < .001) and cancer (*P* < .001) (Table 1). The staining pattern throughout the tissue sections was predominantly cytoplasmic though a small portion (<20%) of epithelial cells demonstrated basal membranous staining pattern in normal, benign, and well-differentiated carcinomas (Figure 1).

Stromal cells of all histological groups did not express S3F with the exception of endothelial cells that, together with positive control placental tissues, expressed S3F in a cytoplasmic and membranous localization (Figure 1, arrow), thus providing in our cohort an internal positive control for S3F expression. Immunostaining was not observed in negative control samples (Figure 1, inset).

### 3.2. NP-2 Expression Decreases with Epithelial Ovarian Cancer Progression

**Figure 3:**
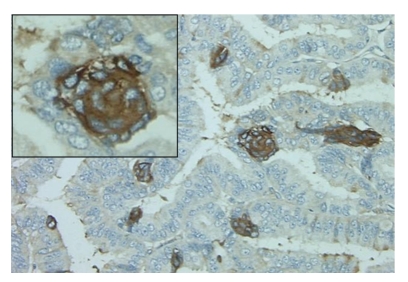
NP-2 expression occurs in distinct clusters of tumor cells. Representative illustration of NP-2 expression in well-differentiated serous adenocarcinoma (WD). Original magnification is 100× and inset is 200×.

NP-2 was generally weakly expressed in all histological groups but the proportion of positive epithelial cells significantly decreased with tumor progression. The expression of NP-2 in 33% (4/12), 20% (2/10), 50% (2/4), 33% (2/6) of N, Cy, WD, and PD was generally weak (Figures 1 and 2) and with no significant statistical difference. In contrast to normal ovaries where 71.4% of OSE positively expressed NP-2, the percentage of positive epithelia was significantly lower in Cy, WD and PD where only 48.8%, 19.2%, and 17.1% were positive, respectively (*P* < .001) (Table 1). The overall staining pattern was cytoplasmic and membranous in all histological groups (Figure 1). Interestingly, most cells expressing NP-2 in the examined WD carcinomas were localized in highly distinctive clusters within the tissue specimens of early stage compared to late stage (Figure 3). 

 In contrast to epithelial cells, over 90% of stromal cells in normal ovaries strongly expressed NP-2 (Figure 1). Similar to normal tissue, stromal cells in Cy, WD, and PD tissues expressed NP-2, however, the level of expression was moderate (Figure 1). Like S3F, all endothelial cells within the stroma and positive control placental tissues expressed NP-2 immunostaining.

### 3.3. NP-1 Expression Increases with Epithelial Ovarian Cancer Progression

In contrast to S3F and NP-2, the overall expression of NP-1 increased significantly with tumor progression. Most (93.8%, 30/32) of the tissues examined expressed NP-1 (Figure 2). The overall staining intensity of NP-1 in N and Cy sections ranged from weak, 58% (7/12) and 60% (6/10), to moderate, 25% (3/12) and 30% (3/10), respectively (Figures 1 and 2, Table 1). In contrast, the vast majority of cancerous tissues, 75% (3/4) of WD and 83% (5/6) of PD samples, strongly expressed NP-1 (Figures 1 and 2); however, no differences were observed as function of stage. The percentage of positive epithelial cells also significantly increased as 80.5%, 86.5%, and 100% of epithelia were positive for NP-1 in N, Cy and cancer tissues, respectively, (Table 1).

Most stromal cells in N and Cy tissues expressed NP-1, although the staining intensity was less than for NP-2. Stromal staining was less in cancerous than in N and Cy tissues (not shown).

### 3.4. S3F, NP-2, and NP-1 Expression is Elevated in Inclusion Cysts, Paraovarian Cysts, and Fallopian Tube Epithelium

**Figure 4:**
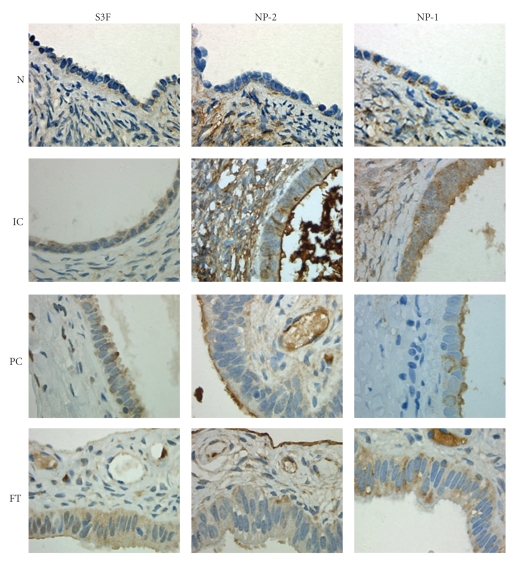
S3F expression is elevated in inclusion cysts, paraovarian cysts, and fallopian tubes. Representative illustrations of immunohistochemical staining of normal ovary (N), inclusion cyst (IC), paraovarian cyst (PC), and fallopian tube (FT) for S3F, NP-2, and NP-1. Original magnification is 400×.

**Figure 5:**
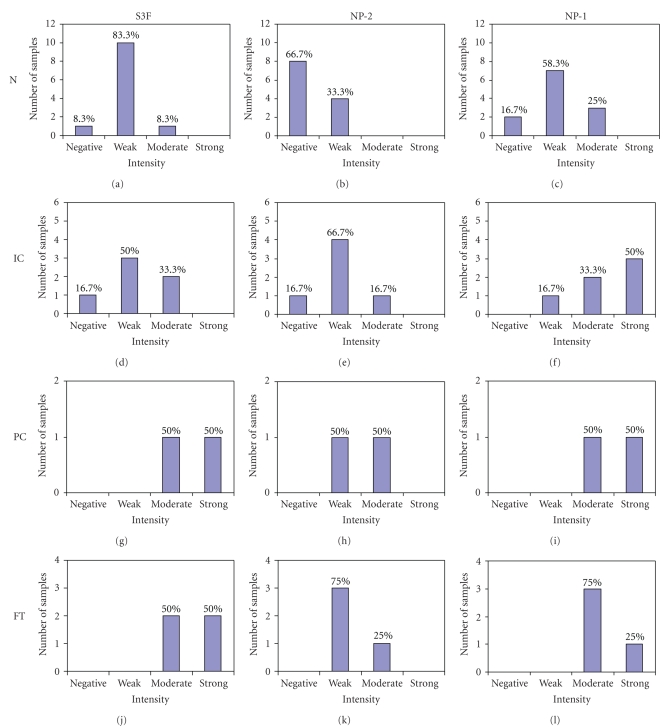
Graphical depiction of S3F, NP-2, and NP-1 expression in inclusion cysts, paraovarian cysts, and fallopian tubes compared to normal ovaries. Immunohistochemically stained sections of normal ovary (N), inclusion cysts (IC), paraovarian cysts (PC) and fallopian tubes (FT) were evaluated for staining intensity and designated as negative, weak, moderate, or strong following staining with antibodies directed against S3F, NP-2, and NP-1.

Given the uncertain cellular origin of EOC, coelomic versus extrauterine Müllerian, we also evaluated the immunohistochemical expression of S3F, NP-2, and NP-1 in ICs, PCs, and FT tissues. We found an elevated staining intensity and percentage of epithelial cells expressing S3F and its receptors in ICs, PCs, and FT sections when compared to normal ovarian and cancerous tissues (Figures 4 and 5, Table 1). In contrast to WD and PD tissues where only 21.6% and 29.5% of the epithelium were positive, 100% of the epithelium lining the ICs expressed S3F (Table 1). All PCs and FT epithelia expressed S3F either moderately or strongly (Figures 4 and 5, Table 1). Similar to the normal ovary, only endothelial cells, but no other surrounding stromal cells expressed S3F. 

 NP-2 expression but not intensity was comparable to S3F in epithelial cells of ICs and PCs (Figures 4 and 5, Table 1). In contrast to WD and PD where only 19.2% and 17.1% of the epithelial cells were positive for NP-2, respectively, 100%, 85.7%, and 72.2% of the epithelia lining ICs, PCs, and FT, respectively, were positive (Table 1). In contrast to the strongly staining stromal cells of the normal ovary, weak NP-2 stromal staining was found in FT and PCs. All endothelial cells were strongly immunoreactive for NP-2. 

 Epithelial expression of NP-1 in ICs, PCs, and FT was universal (Table 1) and similar to cancerous tissues; in contrast to normal ovaries, 50%, 50%, and 25%, respectively of IC, PC, and FT epithelia exhibited strong NP-1 staining (Figures 4 and 5). Similar to NP-2, stromal cells displayed negative to weak NP-1 expression while all endothelial cells were positive.

## 4. Discussion

Loss or delocalization of S3F has been shown to correlate with advanced tumor stage in lung cancer [21, 22]. In this study, we sought to determine the clinical relationship between S3F and epithelial ovarian cancer progression. Overall, we observed a significant decrease in both intensity and frequency of S3F staining with EOC progression. Although, tumors of high grade and advanced stage expressed the least amount of S3F, tumor grade was the only parameter that indicated a significant relationship between S3F expression and EOC progression in this initial cohort of patients. Levels of semaphorin 3A have also been reported to be significantly reduced in advanced EOC and metastases [23]. Taken together, these findings suggest that the loss or deregulation of semaphorin signaling may play an important role in EOC progression and support a tumor suppressor function for this molecule [17]. 

 In contrast, the S3F receptors NP-2 and NP-1 have been reported to be over-expressed in some cancers, including ovarian cancer [22, 23]. In agreement with previous reports, we found that the staining intensity and percentage of epithelium expressing NP-1 significantly increased with EOC progression and predominantly cytoplasmic. However, we found that NP-2 expression decreased with EOC progression. Differences in these results compared to other reported findings may reflect methodological differences in sample preparation, scoring of immunostaining, and case distribution. Interestingly, we observed prominent staining of NP-2 in isolated, but highly distinct clusters of tumor cells in early stage and low-grade (WD) ovarian cancer tissues similar to that described by Brambilla et al. [21] in non-small cell lung cancer. These observations, in addition to the cytoplasmic localization of receptors we observed and previously reported in both lung and ovarian cancers [22, 23], further support a role for a S3F-NP pathway in epithelial cell adhesion and/or migration. 

 Carcinomas arising from the ovary, fallopian tube, and peritoneum have histological and clinical similarities [3]. Histological similarities with epithelia lining ICs, PCs, and FT have also been documented [3] and explained on the basis of common coelomic or Müllerian system origin [4]. In the present study, while there was only weak expression of S3F and NP-2 in EOC, OSE, and IC there was strong expression of NP-1 in FT, PC, and EOC. This shared phenotype indirectly supports a common Müllerian origin for epithelial ovarian cancer. Given the slightly younger premenopausal age of normal individuals compared to the peri- to postmenopausal age of benign and ovarian cancer patients, a potential contribution of menopausal status on S3F expression cannot be ruled out. Although in this initial series, there was no noticeable difference in S3F expression among normal specimens, additional studies are needed to further evaluate independency from hormonal status. 

 In conclusion, our data suggests that the S3F-NP pathway may be deregulated in EOC pathogenesis. Further investigation of S3F and its receptors in epithelial ovarian cancer is warranted to delineate the molecular pathway(s) by which such deregulation may promote tumor progression and, if so, provide novel molecular targets for therapeutic intervention.

## Figures and Tables

**Table 1:** Epithelial expression of S3F and NP-2 decreases while NP-1 increases with ovarian epithelial tumor progression.

	S3F	NP-2	NP-1
N	67.5 ± 1.6	71.4 ± 3.0	80.5 ± 1.4
Cy	42.9 ± 2.3	48.8 ± 2.1	86.5 ± 1.8
	*P ≤ .001 ⋄ *P* ≤ .05	*P ≤ .001 ⋄ *P* ≤ .001	*P ≤ .001 ⋄ *P* ≤ .001
WD	21.6 ± 3.9	19.2 ± 4.4	100
	**P ≤ .001 ∘ *P* ≤ .05	**P ≤ .001 ∘ *P* ≤ .001	**P ≤ .001 ∘ *P* ≤ .001
PD	29.5 ± 1.5	17.1 ± 2.3	100
	**P ≤ .001 ∘ *P* ≤ .05	**P ≤ .001 ∘ *P* ≤ .001	**P ≤ .001 ∘ *P* ≤ .001
IC	100	100	100
PC	100	85.7 ± 4.5	82.5 ± 2
FT	100	72.2 ± 3.6	100

*N versus Cy **N versus WD+PD ⋄N + FT versus Cy ∘N + FT versus WD + PD. Abbreviations: normal (N), serous cystadenoma (Cy), well-differentiated serous adenocarcinoma (WD), poorly differentiated serous adenocarcinoma (PD), inclusion cyst (IC), paraovarian cyst (PC), and fallopian tube (FT). Data represent the average percent of positive epithelium expressing S3F, NP-2, and NP-1 ± SEM.
